# Supplementary material for: Is the Life History Flexibility of Cold Desert Annuals Broad Enough to Cope with Predicted Climate Change? The Case of Erodium oxyrhinchum in Central Asia
Source: Biology (Basel). 2021 Aug 16;10(8):780. doi: 10.3390/biology10080780 (PMC8389623; doi:10.3390/biology10080780)
Supplement: Supplementary file 1 [file biology-10-00780-s001.zip › biology-1291535-supplementary.pdf]

Supplemental tables

Supplemental Table S1. Pearson correlations among life history traits of spring germinating plants (SG) of *Erodium oxyrhinchum* in wet springs.

	Ht	Rl	Nl	La	Nb	Ib	ArO	AsT	AlE	ArE
**Ht**	1									
**Rl**	.330**	1								
**Nl**	.757**	.499**	1							
**La**	.493**	0.046	.651**	1						
**Nb**	.612**	.499**	.788**	.550**	1					
**Ib**	.901**	.320**	.862**	.696**	.687**	1				
**ArO**	.762**	.286**	.814**	.818**	.665**	.927**	1			
**AsT**	.870**	.302**	.848**	.677**	.677**	.975**	.896**	1		
**AlE**	.673**	.395**	.878**	.817**	.729**	.859**	.884**	.841**	1	
**ArE**	.900**	.219*	.664**	.426**	.503**	.890**	.731**	.820**	.563**	1

Ht, height; Rl, root length; Nl, number of leaves; La, leaf area; Nb, number of branches; Ib, individual biomass; ArO, allocation of biomass to roots; AsT, allocation to stems; AlE, allocation of biomass to leaves; ArE, allocation of biomass to reproduction. N = 128, *P <0.05, **P <0.01.

Supplemental Table S2 Pearson correlations among life history traits of autumn germinating plants (AG) of *Erodium oxyrhinchum* in wet springs.

	**Ht**	**Rl**	**Nl**	**La**	**Nb**	**Ib**	**ArO**	**AsT**	**AlE**	**ArE**
**Ht**	1									
**Rl**	0.228	1								
**Nl**	.805**	.393**	1							
**La**	.416**	.595**	.606**	1						
**Nb**	.637**	.552**	.667**	.829**	1					
**Ib**	.909**	.320**	.900**	.589**	.668**	1				
**ArO**	.849**	.458**	.869**	.665**	.736**	.940**	1			
**AsT**	.897**	.280*	.897**	.535**	.604**	.986**	.921**	1		
**AlE**	.644**	.537**	.851**	.870**	.775**	.836**	.874**	.812**	1	
**ArE**	.901**	0.107	.729**	.295*	.489**	.902**	.768**	.868**	.540**	1

Ht, height; Rl, root length; Ln, number of leaves; La, leaf area; Bn, number of branches; Ib, individual biomass; ArO, allocation of biomass to roots; AsT, allocation of biomass to stems; AlE, allocation of biomass to leaves; ArE, allocation of biomass to reproduction. N =64, *P <0.05, **P <0.01.

Supplemental Table S3 Pearson correlations among life history traits of spring germinating plants (SG) of *Erodium oxyrhinchum* in dry springs.

	**Ht**	**Rl**	**Nl**	**La**	**Nb**	**Ib**	**ArO**	**AsT**	**AlE**	**ArE**
**Ht**	1									
**Rl**	0.087	1								
**Nl**	.241*	.279*	1							
**La**	.388**	0.162	.241*	1						
**Nb**	-0.044	-0.048	0.04	0.195	1					
**Ib**	0.246	0.083	.330**	.374**	.331**	1				
**ArO**	0.203	0.332	0.244	0.028	0.247	.630**	1			
**AsT**	.536**	.533**	.351*	.707**	.353*	.853**	0.278	1		
**AlE**	.617**	.618**	.412**	.701**	0.264	.808**	0.167	.933**	1	
**ArE**	0.291	0.343	.446**	.382*	.389**	.767**	.448**	.439**	.415**	1

Ht, height; Rl, root length; Ln, number of leaves ; La, leaf area; Bn, number of branches; Ib, individual biomass; ArO, allocation of biomass to roots; AsT, allocation of biomass to stems; AlE, allocation of biomass to leaves; ArE, allocation of biomass to reproduction. N = 128, *P <0.05, **P <0.01.

Supplemental Table S4 Pearson correlations among life history traits of autumn germinating plants (AG) of *Erodium oxyrhinchum* in dry springs.

	**Ht**	**Rl**	**Nl**	**La**	**Nb**	**Ib**	**ArO**	**AsT**	**AlE**	**ArE**
**Ht**	1									
**Rl**	.242*	1								
**Nl**	0.088	0.137	1							
**La**	.466**	.337*	-0.054	1						
**Nb**	.318**	0.205	.277**	0.142	1					
**Ib**	.287**	0.11	.449**	-0.119	.229*	1				
**ArO**	0.031	0.042	.400**	-0.064	0.115	.394**	1			
**AsT**	.293**	0.05	.387**	-0.13	0.148	.885**	.288**	1		
**AlE**	0.015	0.207	.613**	-0.176	0.132	.694**	.371**	.480**	1	
**ArE**	.390**	0.084	-0.009	0.004	0.086	.612**	0.206	.567**	0.142	1

Ht, height; Rl, root length; Ln, number of leaves; La, leaf area; Bn, number of branches; Ib, individual biomass; ArO, allocation of biomass to roots; AsT, allocation of biomass to stems; AlE, allocation of biomass to leaves; ArE, allocation of biomass to reproduction. N =128, *P <0.05, **P <0.01.
